# DNA damage responses in HCMV infection: emerging mechanisms and outstanding questions

**DOI:** 10.1128/jvi.00376-26

**Published:** 2026-06-16

**Authors:** Mamata Savanagouder, Pierce Longmire, Ahmed Hassan, David McKinzey, Rachel Dansereau, Giovanni Bosco, Felicia Goodrum

**Affiliations:** 1 Department of Microbiology and Immunology, Geisel School of Medicine at Dartmouth, Lebanon, New Hampshire, USA; 2 Division of Protective Immunity, Children’s Hospital of Philadelphia, Philadelphia, Pennsylvania, USA; 3 Department of Pathology and Laboratory Medicine, Perelman School of Medicine, University of Pennsylvania 14640, Philadelphia, Pennsylvania, USA; 4 Department of Molecular and Systems Biology, Geisel School of Medicine at Dartmouth, Hanover, New Hampshire, USA; 5 BIO5 Institute, The University of Arizona 8041, Tucson, Arizona, USA; Universiteit Gent, Merelbeke, Belgium

**Keywords:** DNA damage and repair, human cytomegalovirus, herpesvirus, DNA replication, DNA viruses

## Abstract

The cellular DNA damage response (DDR) is essential for maintaining genome integrity in the face of exogenous assault, as well as errors and breaks introduced during DNA replication. The DDR is a complex network of cellular processes that detects the type and severity of DNA damage and coordinates downstream actions. These include activating specific DNA repair pathways, including cell cycle arrest to allow time for repair, and—if the damage is irreparable—triggering apoptosis to eliminate cells with compromised genomes. DNA viruses have evolved multiple strategies to evade, suppress, or even hijack host DDR factors to promote their own replication. In the absence of such countermeasures, the activation of the host DDR may rapidly detect viral genomes as aberrant DNA, and restrict viral infection. Paradoxically, many DNA viruses depend on many aspects of the host DDR to ensure efficient replication of their genomes. Here, we focus on human cytomegalovirus (HCMV), a beta-herpesvirus with a large complex double-stranded DNA genome, and its relationship with host DDR. Although HCMV infects most of the human population, fundamental gaps remain in understanding how viral replication modulates a robust cellular DDR or recruits it for its own replication. Moreover, the roles of DDR in restricting viral DNA (vDNA) replication, promoting entry into latency, stabilizing latent genomes during cellular proliferation, and promoting vDNA synthesis following infection or reactivation from latency remain poorly understood.

## INTRODUCTION

**Fig 1:**
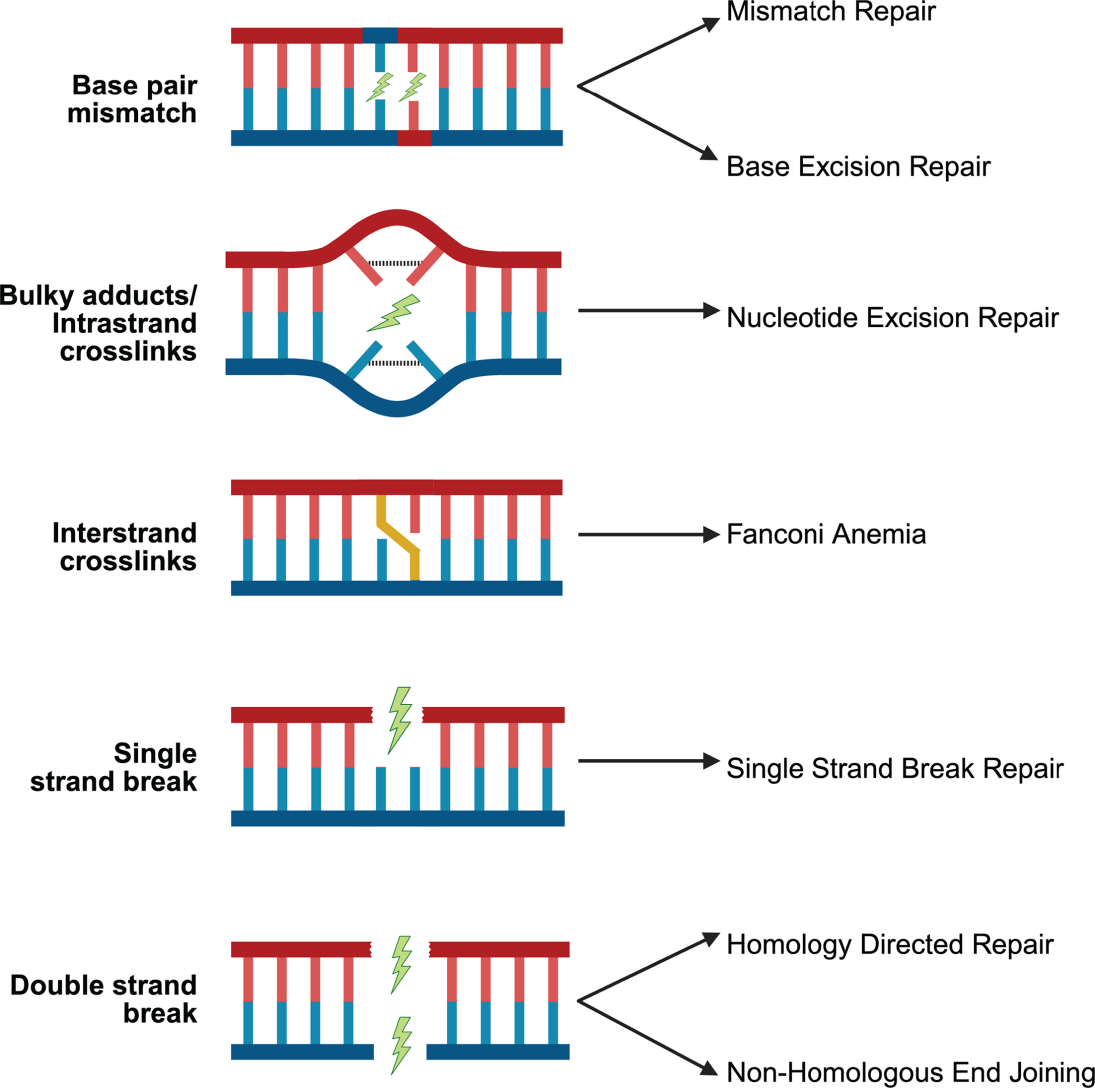
DNA lesions and repair pathways. (Top to bottom) Mispaired DNA bases can be replaced with correct bases via mismatch repair (MMR) or base excision repair (BER), which can also address single damaged bases. Larger bulky DNA adducts and intrastrand crosslinks can cause significant distortions to the DNA helix. These types of lesions are often corrected by nucleotide excision repair (NER), which removes oligonucleotides containing the damaged bases. Another type of crosslink, interstrand crosslinks (ICLs), can be repaired by the Fanconi anemia (FA) pathway. Even more complex forms of damage involve breaks in the DNA backbone. Single-strand DNA breaks (SSBs) are repaired by single-strand break repair (SSBR). More deleterious double-strand breaks (DSBs) involve breaks in both complementary strands and can be repaired by multiple pathways, including non-homologous end joining (NHEJ) and homology-directed repair (HR). Created in https://BioRender.com.

The DNA damage response (DDR) is essential for maintaining the integrity of cellular genomes. DNA damage can result from environmental insults such as oxidative stress, mutagens, UV and ionizing radiation, pathogens, as well as during normal cellular proliferation. In human cells, an estimated 10–50 double-strand breaks (DSBs) (1) and 1–3 single nucleotide variants (SNVs) arise within coding regions (2, 3) every cell cycle. Consequently, because every cell division is a possible mutagenic event, each human will have produced about 10^17^ DSBs throughout a typical lifespan (4). Given this burden, it is not surprising that robust cellular DNA damage surveillance, detection, and response mechanisms have evolved to maintain genome stability by promoting downstream repair or apoptosis when damage is irreparable. Defects in the DDR, or downstream repair pathways, are associated with multiple disorders including premature aging, neurological disorders, skin sensitivities, and cancers (5–8). To counteract the deleterious effects of DNA damage, cells have evolved a myriad of DNA repair pathways with specificity for addressing certain types of lesions. DNA lesions are detected based on the distinct DNA structure they present to damage surveillance proteins that constantly patrol the genome (Fig. 1). For example, mismatched base pairs, stalled replication forks, single-strand DNA breaks (SSBs), and DSBs each present unique structures, which recruit unique ensembles of the DDR and repair proteins to the damaged sites (Fig. 1).

The DDR is also activated by microbial infections, particularly infections by viruses with DNA genomes (9–12). The earliest step of virus infection, entry, introduces the viral genome into the cell, which is recognized as foreign nucleic acids (13). Nicks and gaps in the viral genome, as well as free double-stranded ends of linear genomes, activate a DDR that triggers DNA repair pathways (14, 15). If infection is successfully established, viral genome synthesis provides another opportunity for DDR activation as viral DNA (vDNA) is vulnerable to the same error-prone DNA synthesis and repair events as cellular DNA (13). As a significant host defense system, activation of a DDR can serve to rapidly detect and limit viral infection, as several factors involved in the DDR have known roles in immune activation (16, 17). Crosstalk between the DDR and innate immune system also activates multiple host pathways to eliminate or restrict pathogens (10). For example, ATM stimulates interferon responses, activates NFκB, and activates p53, which will associate with IFI16 and STING, and unrepaired DNA can activate IFN responses in ATM-deficient cells. Unsurprisingly, viruses have evolved multiple tactics to evade, suppress, or even hijack these host defenses to promote their own replication, as discussed in detail below.

## DDR PATHWAYS—AN OVERVIEW

Factors involved in the DDR are typically divided into three major classes: sensors, transducers, and effectors. The first step of DDR activation is recognition of damage or replication stress by sensors that physically bind to DNA structures (e.g., base pair mismatch, stalled replication fork, breaks, etc.). DDR sensor proteins at sites of DNA lesions activate kinases locally to transduce signals and activate specialized effectors to address specific classes of DNA damage. Effector proteins, including a variety of DNA polymerases, are the machines that restore damaged DNA to its undamaged state. Simple lesions arising from misincorporated bases and bases with small chemical modifications are repaired by the mismatch repair (MMR) and base excision repair (BER) pathways, respectively (18). Nucleotide excision repair (NER) is recruited to address larger lesions, such as pyrimidine dimers and intrastrand crosslinks (19). Repair of interstrand crosslinks (ICLs) is mediated by proteins in the genetic complementation group for the hereditary disorder Fanconi anemia (FA) (20). Moving to more complex lesions, SSBs are addressed in the single-strand break repair (SSBR) pathway. Finally, nonhomologous end joining (NHEJ) and homology-directed recombination (HR) are two major pathway choices for repair of highly toxic DSBs and will be discussed in further detail below (21). DNA lesions are not always repaired immediately, particularly if damage is encountered during replication. Replication fork progression can be maintained by tolerance mechanisms known as lesion bypass, which include template switching (TS) and translesion synthesis (TLS) pathways (22, 23). While this list is neither exhaustive nor definitive—especially considering that multiple pathways may be employed for a single lesion—it highlights some of the major effector pathways of the DDR. The pathways employed by specific lesions are depicted in Fig. 1.

**Fig 2:**
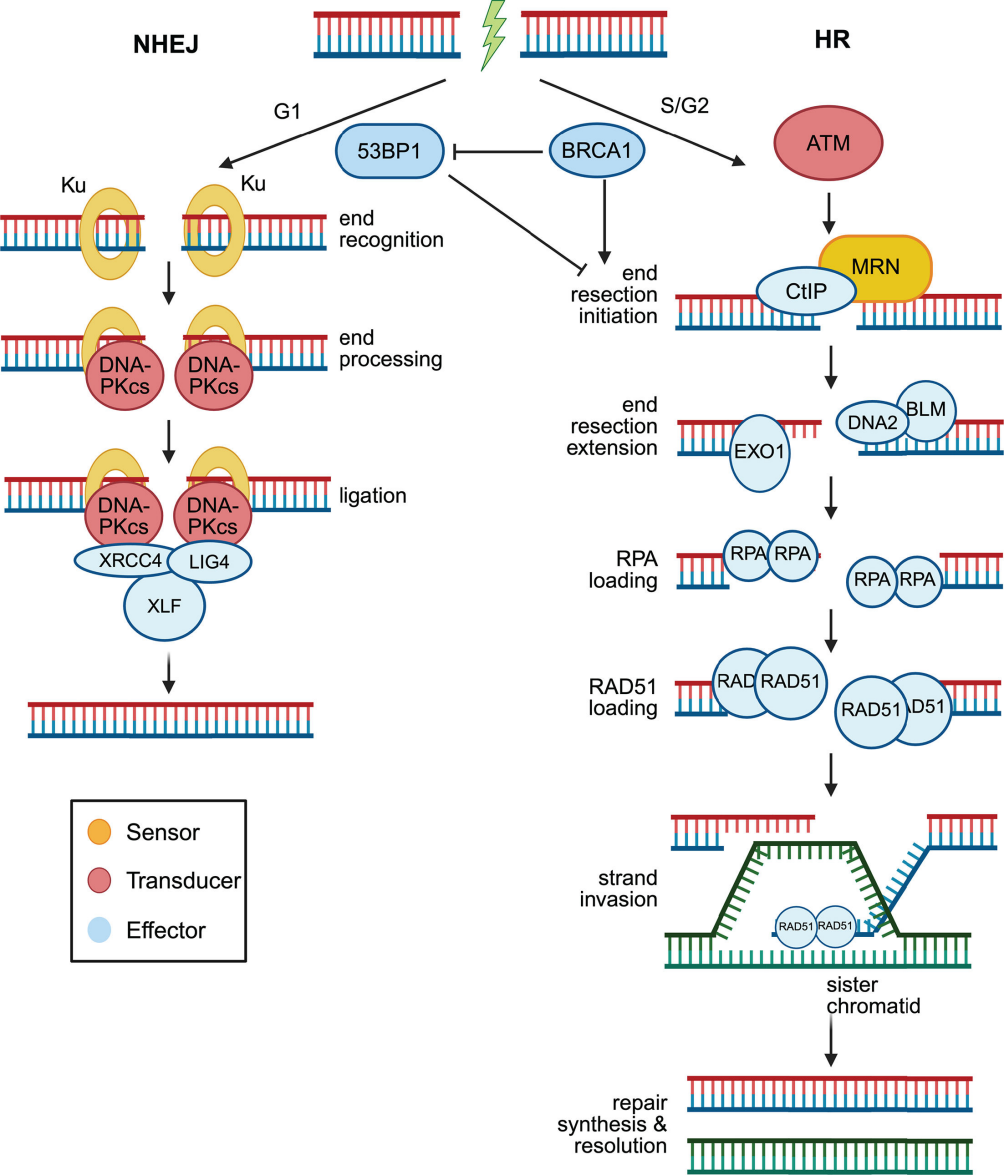
DSB repair by NHEJ and HR pathways. The decision to repair DSBs by the NHEJ or HR pathway is mediated by the proteins, 53BP1 and BRCA1. During the G1 phase of the cell cycle, 53BP1 is associated with chromatin, which limits resection of the DSB ends and favors repair by NHEJ. In this pathway, Ku70/80 binds DSB ends and recruits DNA-PKcs for end processing. DNA-PKcs then recruits various proteins, including key factors LIG4, XRCC4, and XLF, to ligate the broken ends. Alternatively, during the S and G2 phases of the cell cycle, BRCA1 expression can inhibit 53BP1 interaction with chromatin, allowing for DSB end resection. The sensor and kinase ATM activate CtIP and MRE11 (of the MRN complex) to initiate the process. End resection is further carried out through the activity of BLM, DNA2, and EXO1 proteins. Following this, RPA binds and stabilizes ssDNA and is later displaced by RAD51 to promote homology-directed strand invasion of the available sister chromatid. The sister chromatid is used as a template for accurate synthesis, resolving repair of the break. Created in https://BioRender.com.

The NHEJ pathway is active during all phases of the cell cycle and, unlike HR, does not require a homologous template for repair. NHEJ mediates direct ligation of broken DSBs and is thereby inherently more error-prone. NHEJ is initiated when Ku70/80 proteins recognize and bind DSBs, recruiting the transducer, DNA-dependent protein kinase catalytic subunit (DNA-PKcs), to the site of DSB, forming the complete DNA-PK complex. These events trigger end-processing by nucleases and polymerases (pol), including Artemis, Pol μ, and Pol λ, to ensure the ends are compatible for ligation. Ligation of the DNA ends is then carried out by the XRCC4-LigIV-XLF complex (Fig. 2) (21, 24, 25). NHEJ can repair breaks that restore the original DNA sequence, or it often can lead to small (<20) nucleotide deletions or insertions.

HR is a high-fidelity mechanism of DSB repair that is primarily active during S and G2 phases of the cell cycle when a sister chromatid is available to be used as a template. HR is regulated by two signal transducers, ataxia telangiectasia mutated (ATM) and ataxia telangiectasia and Rad3-related (ATR) kinases. The ATM-mediated pathway is initiated when the key sensor, the MRN (Mre11-Rad50-NBS1) complex, recognizes and binds to the broken ends of the DNA and activates ATM (26). ATM autophosphorylates and phosphorylates various substrates, such as H2AX and NBS1, to amplify DDR signaling and recruit downstream effector proteins. This leads to extensive 5′ to 3′ end resection, generating regions of single-stranded DNA (ssDNA). Replication protein A (RPA) rapidly coats the exposed ssDNA, protecting it from degradation and folding into secondary structures. Rad51 recombinase subsequently replaces RPA by the coordinated action of the BRCA1-BRCA2-PALB2 complex and mediates homology search and strand invasion of a homologous target (27, 28). Subsequent recruitment of DNA polymerases uses homologous templates to initiate synthesis and enable completion of DNA repair (Fig. 2) (29). In addition to promoting repair, ATM activation leads to phosphorylation of checkpoint kinase 2 (chk2) and p53 to mediate cell cycle arrest at the G1/S checkpoint or induce apoptosis in cases where repair is irreparable (21, 30–32). Unlike DNA-PK and ATM, which are activated by the sensors Ku70/80 and MRN respectively, ATR is activated by its recruitment to RPA on ssDNA via the ATR interacting protein (ATRIP). Long stretches of ssDNA generated at stalled replication forks and during end resection of DSBs in the ATM-mediated DDR may initiate an ATR-mediated response. Complete activation of ATR requires several other proteins, such as the 9-1-1 (Rad9-Rad1-Hus1) complex, Rad17, and topoisomerase B1 (TOPB1). Once activated, ATR phosphorylates checkpoint kinase 1 (chk1), leading to cell cycle checkpoint activation to allow for repair to occur before progression into G2/M phase (21, 31, 32). While these are defined as distinct pathways, much interplay exists as DNA-PK, ATM, and ATR can activate the same substrates, such as H2AX and checkpoint kinases (33).

## DNA VIRUSES AND HOST DDR

DNA viruses comprise a diverse group of pathogens with genomes that span a wide range of sizes and organizational complexity, employing an equally varied set of replication strategies. Viral genomes can be comprised of linear ssDNA, such as that of parvoviruses, or dsDNA arranged in linear or circular arrangements, such as herpesviruses (12). Adeno-associated viruses (AAV) represent the smallest DNA viruses with a genome of ~5 kb (34), while herpesviruses are some of the largest studied DNA viruses to infect animals with genome sizes ranging from 110 to 240 kb (35). Regardless of genome size or arrangement, these DNA viruses commonly activate DDR sensors and transducers upon infection, but to different extents and effects (12). For example, AAV infection has been shown to activate both ATM and ATR signaling (36, 37). Human papillomaviruses (HPVs) and polyomaviruses that have circular dsDNA genomes also activate both ATM and ATR signaling upon infection (38, 39). Herpesviruses with large, linear dsDNA genomes use distinct strategies in their regulation of DDR pathways. Herpes simplex virus 1 (HSV-1) activates ATM and largely inhibits ATR but uses specific ATR-specific downstream effectors (40–43), while Kaposi’s sarcoma-associated herpesvirus (KSHV) activates components of both ATM and ATR pathways (44, 45).

While the DDR may be activated as a response to infection, many viruses hijack host DDR machinery for vDNA synthesis. For example, HPV recruits many host DDR factors to sites of vDNA replication and relies on host DNA polymerases for replication (46). Even the poxvirus, vaccinia, which replicates its genome in the host cell cytoplasm, activates ATR signaling during infection and relies on some host machinery for efficient replication (47). Specific host factors are also recruited to HSV-1 genomes upon nuclear entry, likely in response to nicks and gaps within the genome that could be recognized as a template for repair (14, 15). However, while some host machinery is exploited for replication, the HSV-1 encoded immediate early protein and E3 ubiquitin ligase, ICP0, selectively degrades host factors involved in intrinsic immune and DDR responses to infection, including at least two E3 ligases that repress transcription of incoming genomes (48), DNA-PKcs (49, 50), and components of nuclear domain 10 bodies (ND10s) (51, 52). Although some disagreement exists (53), ICP0 mediates degradation of DNA-PK, which is proposed to prevent genome circularization—the genome confirmation associated with latency—thereby promoting replication by maintaining the genomes in a linear, replication-competent state (54, 55). In KSHV infection, both DNA-PK and ATM contribute to circularizing viral genomes for latency, and perturbation of these DDR factors interferes with the efficiency of latency establishment (55). The complexity of virus interactions with host machinery is especially evident in viruses that establish persistent infections within their host, where selective recruitment of host machinery might impact decisions to replicate or enter into a latent state (56). For example, in the case of HSV-1, establishment of latency requires the rapid circularization of the incoming genome, a process that is dependent on DNA-PK, as well as transcriptional repression of viral gene expression. Both processes are antagonized by ICP0, which directly targets DNA-PK and the E3 ubiquitin ligases RNF8 and RNF168, key regulators of histone ubiquitination and chromatin-based DNA damage responses (54, 55, 57). Collectively, these findings suggest that ICP0 expression inhibits HSV-1 latency by disrupting DNA-PK-dependent genome circularization and chromatin-mediated repression.

Virus hijacking of host DDR machinery often comes with the collateral damage of detrimental effects on host genome integrity. This is especially true for oncogenic viruses like HPV, which dysregulate cellular growth through inhibition of the tumor suppressors, p53 and retinoblastoma protein (pRb) (58–60). Further, KSHV induces DSBs but inhibits DSB repair machinery (61). Persistent viral infections have also been associated with induction of micronuclei (62), dysregulated formation of DNA:RNA hybrids (63), and various chromosomal abnormalities (64, 65), all of which contribute to host genome instability.

Although many viruses activate a robust host DDR, it is often unclear whether this represents a host defense response aimed at limiting viral replication or whether the viruses selectively co-opt components of the DDR to promote their replication. Table 1 summarizes host cell DDR responses and likely functions of the DDR in inhibiting or promoting replication of DNA viruses. We note that this is not an exhaustive list, and it is limited to human viral pathogens. Our extended discussion below focuses on the beta-herpesvirus HCMV. Virus-mediated control of the host DDR in genome replication and dictating states of infection is perhaps most poorly understood in the context of HCMV infection. In the following sections, we will review the body of knowledge around HCMV in triggering, controlling, or co-opting host DDR and how it influences infection, with other viruses discussed for comparison and reference.

**TABLE 1:** Host DDR proteins commonly implicated in DNA virus infection

DDR pathway/factor	Typical host outcome	Trigger during infection	Effect on viral replication	Representative DNA viruses
ATM kinase	G_1_/S arrest, checkpoint activation, HR recruitment (21, 30–32)	Incoming viral DNA, replication-associated breaks	Often proviral	HSV-1, HCMV, and HPV activate ATM to support replication compartments (14, 15, 46, 66)
ATR kinase	S-phase checkpoint, fork stabilization (21, 31, 32).	ssDNA, stalled replication forks	Context-dependent, often restrictive	HSV-1 and adenovirus suppress ATR signaling (36, 37, 40–43)
DNA-PK (NHEJ)	Rapid end-joining, genome ligation (21, 24, 25)	Free DNA ends of incoming genomes	Antiviral, prolatency	HSV-1 inhibits DNA-PK activity, viral genome circularization in KSHV and HSV-1 (49, 50, 55)
Homologous recombination (RAD51, BRCA1/2)	High-fidelity repair, recombination (21, 30–32)	Replicating viral DNA, replication stress	Proviral	HSV-1, HCMV, and HPV recruit HR machinery (15, 46, 67, 68)
MRN complex	ATM activation, end resection (26)	Sensing viral DNA ends	Antiviral unless neutralized	Adenovirus and HSV-1 degrade or relocalize MRN (69, 70)
p53 signaling	Cell cycle arrest, apoptosis (21, 30–32)	ATM/ATR activation	Antiviral	HCMV activates p53, HPV E6, Ad E1B inactivate p53 (66, 71–73)
Chk1/Chk2	Checkpoint enforcement (21, 30–32)	DDR kinase signaling	Usually antiviral	HSV-1 activates Chk2 but suppresses Chk1 (15)
PARP1	DNA repair, chromatin remodeling, innate signaling (74)	DNA breaks, oxidative stress, viral DNA	Proviral	modulates cGAS in HSV-1, decreases IFN-1 response in HCMV (75, 76)
γH2AX	Recruitment of repair factors (77)	DSB signaling at viral genomes	Proviral	Enriched in herpesvirus replication compartments (67, 78)
FA pathway	Fork protection (79)	Replication stress, crosslinks	Proviral	HPV exploits FA pathway during differentiation (80)

## HCMV

HCMV, also known as human herpesvirus 5, is a ubiquitous herpesvirus that has co-evolved with its human host to persist in the majority of the global population. Like all herpesviruses, HCMV establishes a lifelong latent infection, and the outcome of infection—replicative or latent—is highly dependent on the cell type infected (81). Cells, such as fibroblasts, stromal cells present within connective tissues, support productive infection and are common models for studying HCMV in a laboratory setting (82). Endothelial and epithelial cells also support HCMV replication, albeit at lower levels, described as chronic or “smoldering” infection (83). Viral latency is a quiescent infection where viral genomes are maintained in the absence of viral genome replication and viral progeny production (81). Hematopoietic progenitor cells (HPCs) and cells of the myeloid lineage (e.g., monocytes) have been described as a major reservoir for latency (84, 85). Changes in cellular differentiation or stress can stimulate the virus to reactivate, re-entering productive replication (86–88). Large gaps exist in our mechanistic understanding of how the HCMV genome is replicated, the conformation of the genome during latency, and how it is maintained during cell division.

## VIRAL GENOME SYNTHESIS

HCMV has a large (~236 kb), double-stranded, linear DNA genome divided into unique short (U_S_) and unique long (U_L_) regions flanked by long (>1 kb) inverted repeat sequences, which allow for inversions of these regions to generate four equimolar isomers of the genome (isomerization) (89). While little is known about HCMV genome isomerization, HSV-1 isomerization is initiated by DSBs within inverted repeats of the genome and occurs through HR (90). HCMV and HSV-1 have similar genome structures, both classified as herpesvirus class E; therefore, it is possible that HCMV employs similar mechanisms for isomerization (91). HCMV encodes functional homologs of eukaryotic DNA replication machinery (Table 2), a feature largely conserved among herpesviruses (92). Although the core viral proteins and a limited number of host factors important to replication of the viral genome have been identified, the mechanisms by which HCMV replicates its genome remain poorly defined.

**TABLE 2:** Viral proteins important in the synthesis of herpesvirus genomes^*a*^

Replication function	HSV-1 (α)	HCMV (β)	KSHV (γ)	Function description	Eukaryotic functional homolog
DNA polymerase (catalytic)	UL30 (93)	UL54 (94)	ORF9 (95)	Viral DNA synthesis	DNA polymerase δ / ε (96)
Polymerase processivity factor	UL42 (93)	UL44 (94)	ORF59 (95)	Tethers polymerase to DNA, increases processivity	PCNA (96)
Helicase	UL5 (93)	UL105 (94)	ORF44 (95)	DNA unwinding at replication fork	Superfamily 1 helicases, such as upf1, pif1, RecB, RecD2 (97–99) MCM7 (100)
Primase	UL52 (93)	UL70 (94)	ORF56 (95)	RNA primer synthesis for lagging strand	Prim1, PrimPol (101)
Helicase-primase accessory	UL8 (93)	UL102 (94)	ORF40/4 (95)	Stabilizes helicase–primase complex	Unknown
ssDNA binding protein	UL29 (ICP8) (93)	UL57 (94)	ORF6 (95)	Stabilizes ssDNA, promotes recombination	Rad52 (102)

^*a*^ Proteins included are encoded by the prototypical virus for the alpha, beta, and gamma subfamilies.

**Fig 3:**
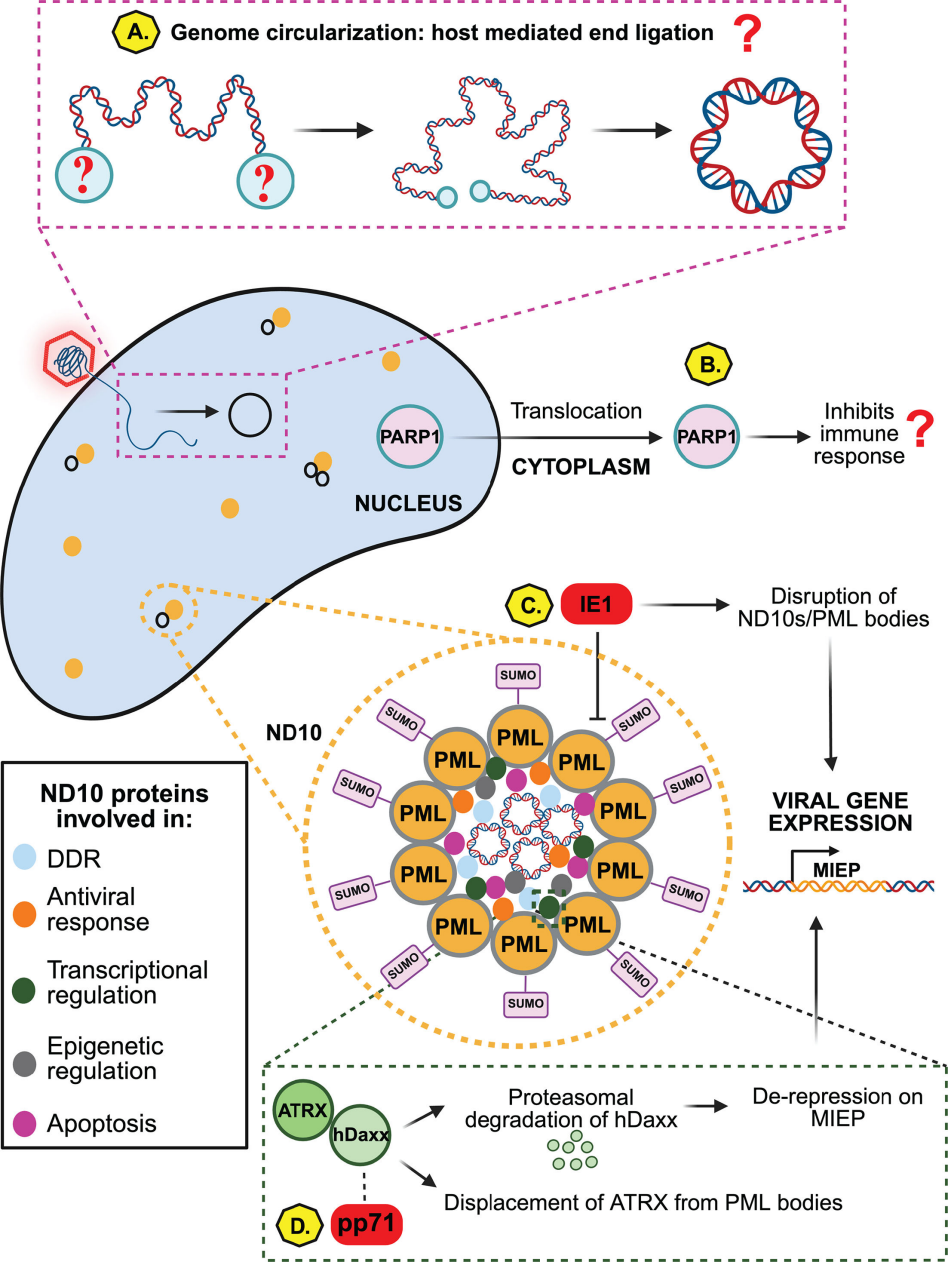
Host DDR protein involvement in establishment of infection. (**A**) The structure of the HCMV genome likely recruits host DNA repair proteins immediately following its entry into the nucleus. Upon extrusion of the viral genome from the capsid into the nucleus, it is possible that the dsDNA ends or nicks and gaps on the genome are recognized as damaged genetic material and recruit host DDR factors. While the structure of the genome during virus replication is not known, it is maintained in latency as a circular episome. The mechanisms important to circularization have not been defined but could be due to NHEJ-mediated ligation of the genome ends. (**B**) PARP1 is activated following infection, perhaps due to presence of nicks and gaps in the viral genome or generation of reactive oxygen species upon nuclear entry. PARP1 translocates to the cytoplasm, where it inhibits the type 1 INF response. HSV-1 similarly induces PARP1 translocation and inhibits IFN through direct inhibition of cGAS. (**C**) Viral genomes localize to PML nuclear bodies, phase- separated nuclear bodies that are composed of various proteins, including PML, hDaxx, SP100, and ATRX. PML nuclear bodies sequester viral genomes and prevent gene expression. To counteract this, IE1 binds the PML protein, preventing its SUMOylation and disrupting host factor recruitment to PML bodies. Through this process, IE1 counteracts a major host antiviral defense, allowing for viral gene expression from the major immediate early promoter (MIEP). (**D**) In addition to IE1-mediated disruption of PML nuclear bodies, the viral tegument protein pp71 promotes proteasomal degradation of hDaxx, displacing ATRX and further disrupting PML bodies to create an optimal nuclear environment for viral gene expression and, presumably, vDNA synthesis. Created in https://BioRender.com.

As observed for other DNA viruses, prior to the onset of genome synthesis, the genome translocates to the nucleus and associates with distinct nuclear bodies known as promeylocytic leukemia (PML) bodies or nuclear domain 10 (ND10s) (Fig. 3). ND10s are dynamic, phase-separated nuclear bodies that sequester a variety of potent regulatory proteins involved in DNA repair, chromatin modification, epigenetic regulation, cell cycle control, and apoptosis, with known roles in oncogenesis (103, 104). The main constituents of ND10s are the chromatin regulators SP100 nuclear antigen (SP100), human death domain-associated protein 6 (hDaxx), and the PML protein itself (105). hDaxx recruits alpha thalassemia/mental retardation X-linked protein (ATRX) to viral genomes and this complex functions as a histone H3.3 chaperone, depositing this H3K9me3-marked variant onto viral chromatin in a replication-independent manner, contributing to the heterochromatinization and silencing of the major immediate-early promoter (MIEP) (106). This DNA replication-independent histone deposition is critical for inhibiting early viral gene expression that would otherwise lead to productive replication. DNA viruses, including HCMV, encode proteins to overcome ND10-mediated restriction, described in more detail in the following sections.

**Fig 4:**
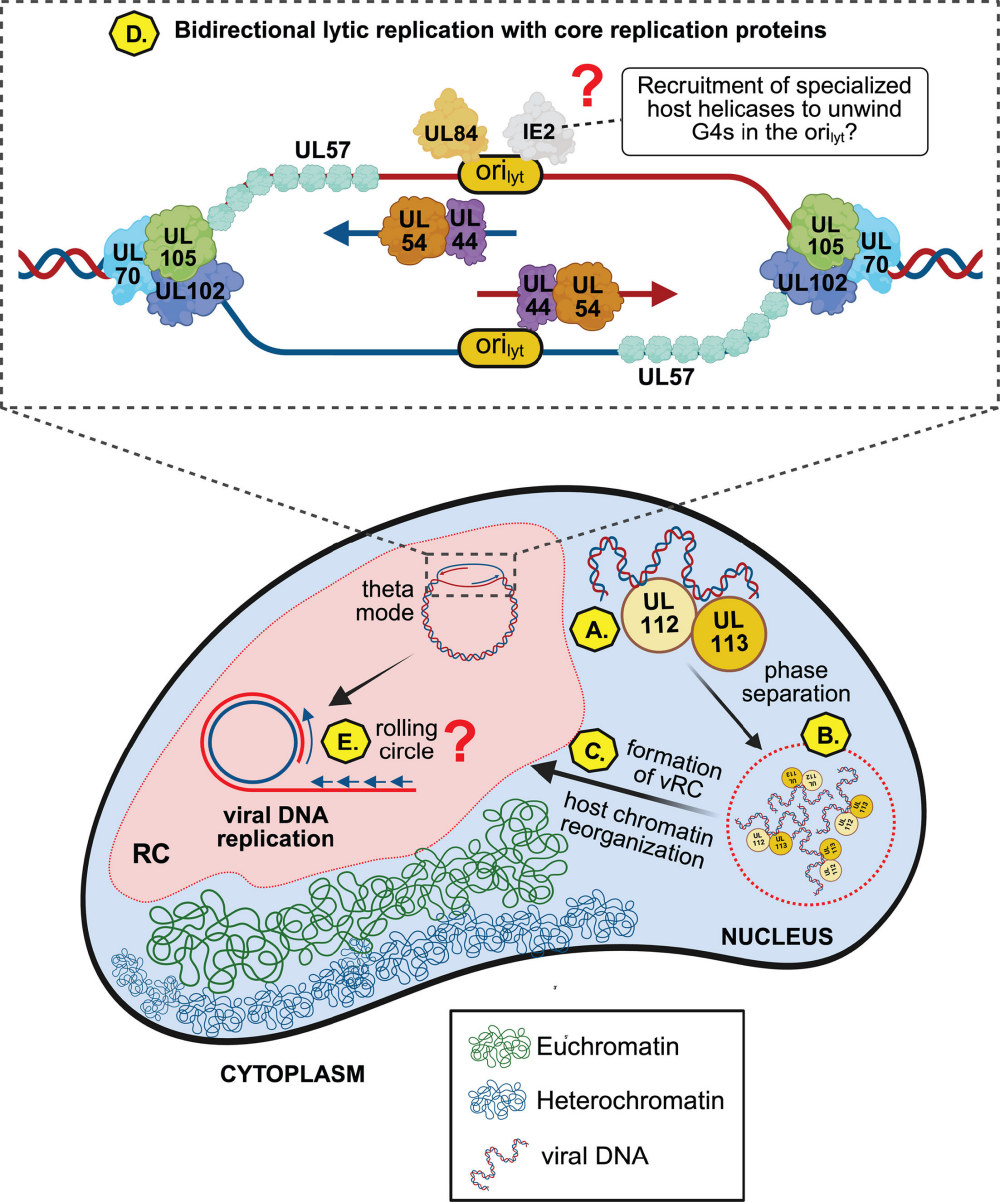
Overview of HCMV genome synthesis. (**A**, lower half) At early times of infection, viral phosphoproteins encoded by the *UL112-113* genes bind viral genomes in the nucleus. These gene products contain intrinsically disordered regions, and (**B**) through interaction with this vDNA, UL112-113 proteins induce liquid-liquid phase separation, forming small pre-replication foci in the nucleus. These foci, which contain viral DNA as well as host and viral proteins, increase in size and complexity as viral DNA is synthesized. (**C**) During this expansion into a full, phase-separated viral replication compartment (vRC, red), host chromatin is rearranged with heterochromatin being pushed to the periphery of the nucleus. (**D**, upper half) Within the vRCs, the genome is presumed to be circularized and thought to undergo a theta mode of DNA replication initially. The viral proteins UL84 and IE2 interact with G4 secondary structures near the origin of replication, oriLyt, which may help to unwind DNA and initiate replication. It is unknown whether other viral or host factors, such as specialized helicases, are required for this process. Genome synthesis occurs bidirectionally from oriLyt and is primarily carried out by viral proteins. UL70, UL102, and UL105 make up the helicase-primase complex, while UL57 binds and stabilizes single-stranded DNA. The viral DNA polymerase, UL54, and its presumptive processivity factor, UL44, facilitate polymerization of nascent DNA strands through leading and lagging strand synthesis. (**E**) Following an initial theta mode of replication, viral DNA synthesis is traditionally thought to switch to a rolling circle mechanism for mass, rapid production of head-to-tail concatemeric genomes; however, this model has been recently challenged by studies in HSV-1 supporting a significant role for recombination-driven replication. Created in https://BioRender.com.

The timing of initiation of HCMV genome replication following infection depends on both cell type and multiplicity of infection and occurs from a complex, bidirectional lytic origin of replication (oriLyt) (Fig. 4) encompassing at least 1,500 bp between UL57 and UL69 genes, an origin of replication considerably larger and more complex than other beta-herpesviruses (94, 107, 108). HCMV contains only a single identified origin, and its disruption abolishes virus replication in laboratory-adapted stains of HCMV (109). Considering that HCMV has only one origin of replication, replication forks would be anticipated to stall while replicating its large and complex 236-kb genome. How HCMV overcomes replication stress associated with fork stalling and completes faithful replication of its complete genome is poorly understood. Further, the extent to which the host DDR is involved in the repair of these stalled replication forks or other damage is unknown. The core replication fork is comprised of six conserved proteins (Table 1): the DNA polymerase (UL54 catalytic subunit and UL44 accessory subunit and presumptive processivity factor), a single-stranded DNA-binding protein (UL57), and the helicase-primase complex (UL105, UL102, UL70) (Fig. 4) (94). UL44 forms a head-to-head dimer in the shape of a C clamp that binds DNA to tether UL54 to the template to stimulate long-chain synthesis (110). Drawing from other herpesviruses, leading and lagging strand synthesis is thought to be coupled and primed by RNA, which must be removed by host or viral factors that have yet to be determined (93, 111).

The HCMV genome is partitioned into nuclear replication compartments (RCs) (Fig. 4). The UL112-113 phosphoproteins (p34, p43, p50, and p84) interact through intrinsically disordered regions to drive liquid-liquid phase separation to form RCs for vDNA synthesis (Fig. 4) (112). Viral and cellular proteins important for DNA synthesis, transcription, and RNA processing are recruited and compartmentalized within RCs for *de novo* synthesis of viral genomes. UL84 binds to the stem-loop region of the oriLyt and may serve to unwind this structure to initiate replication (Fig. 4) (94, 113). UL84, along with IE2, interacts with G-quadruplex secondary structures (G4) in the oriLyt to initiate replication (discussed further in the following section) (Fig. 4) (114). UL114, the viral uracil-DNA glycosylase, interacts with the vDNA polymerase to excise uracil in the genome caused by spontaneous deamination of cytosine or misincorporation (115) for late-phase amplification of viral genomes (116). Further, polarization of the inner nuclear membrane SUN1 proteins is necessary to reorganize actin filaments to spatially segregate vDNA from inactive histones and host DNA for RC formation (112, 117). In addition to actin remodeling, SUN1 and microtubules play a role in large-scale reorganization of the host genome such that viral genomes on the periphery of the RC are in close contact with transcriptionally active regions of the host chromatin while being physically segregated from repressive regions; however, its precise role in infection remains to be understood (Fig. 4) (118).

A rolling circle mechanism has been proposed for vDNA synthesis, starting as a theta form initiating at the oriLyt, which will produce several thousand genome copies per cell by the time the replication cycle is complete (Fig. 4) (94, 119). The rolling circle model for herpesviral DNA replication is supported by the formation of head-to-tail concatemeric genomes and the ability of purified core replication proteins to support the replication of circular molecules of DNA *in vitro* (94, 119–122). However, definitive evidence demonstrating replication of an episome is lacking, and this long-standing model has been recently challenged for HSV-1 (15, 54, 102, 123–126). In HSV-1, genome circularization is not a prerequisite for the initiation of genome replication. ICP0 targets DNA-PK for degradation, thereby preventing genome circularization; circular genomes are observed only in mutant viruses lacking ICP0 (54, 55). These findings suggest that HSV-1 can replicate its genome using a different mode of replication than the proposed theta mode of replication. How this occurs remains to be understood, and if this holds true for all herpesviruses remains to be determined. Increasing evidence suggests that recombination may play a role in replication of HSV-1 replication (15, 102, 123–127). Analysis of replicating HSV-1 DNA using pulse field gel electrophoresis (PFGE) have revealed large amounts of vDNA retained in the well of agarose gels, indicating the presence of complex and highly branched structures that are resistant to electrophoretic migration (54, 128, 129). Whether these structures represent concatemers generated by rolling circle replication or branched structures arising from repair and recombination by the activity of recruited host DDR factors remains unclear. The presence of X and Y structures in electron micrographs and 2D gel electrophoresis of replicative HSV-1 DNA also indicates the presence of highly branched structures (129–131).

HCMV has been reported to circularize upon nuclear entry in replicative infection (119), and it is maintained as a circular episome during latency (132, 133). However, there is little evidence to definitively support the CMV genome forming circles during replication (as discussed above), and detection of terminal restriction fragments suggest CMV genomes replicate as linear molecules (119). A study examining HCMV genome conformation using PFGE also reported the accumulation of high-molecular-weight (HMW) vDNA during infection. While this approach does not distinguish between linear and circular genomes, the authors attributed HMW DNA retained in the well to the presence of circular genomes early in infection and concatemer formation at later times in infection. Notably, irradiation of the HMW DNA did not generate unit length molecules as would be expected if the DNA primarily consisted of concatemers (119). This raises the possibility that, in addition to circular genomes, the HMW DNA may include highly branched replication intermediates, similar to those observed during HSV-1 infection. Taken together, these findings highlight the need for a more thorough analysis of HCMV genome conformation during replication. Application of approaches, such as Gardella gel electrophoresis, would be particularly informative in assessing the extent and functional significance of genome circularization, the nature of replication intermediates, and the role of host DDR pathways in shaping these processes.

## HOST FACTORS RESPONDING TO HCMV INFECTION

Many host factors important to DNA repair and transcription are recruited to RCs during virus replication to regulate genome synthesis and gene expression. The structure of the HCMV genome likely recruits host DNA repair factors to the genome. Extrusion of the genomes from the capsid presents two double stranded DNA ends that likely resemble DNA breaks. While the genome is thought to form an episome for replication, definitive demonstration of this is lacking for HCMV, as discussed above (Fig. 3). However, DNA-PK-mediated circularization of the linear genomes has been demonstrated for KSHV (55), and circular genomes are broadly accepted as the conformation of the genome of all herpesvirus for latency. Additionally, the incoming HCMV genome may consist of nicks and gaps, as has been demonstrated for HSV-1, which could trigger the DDR (14). Consistent with this, PARP1 activation at early times during HCMV infection is attributed to the presence of nicks and breaks in the genome or generation of reactive oxygen species immediately after infection. During HCMV infection, translocation of PARP1 to the cytoplasm and an increased IFN-1 response observed in PARP1 deficient cells suggest an antiviral suppressive role for PARP1 (Fig. 3) (75). A similar phenomenon has been described for HSV-1, where PARP1 also translocates to the cytoplasm and ADP-ribosylates cGAS, inhibiting its DNA-binding activity and subsequent induction of antiviral signaling (76). Translocation of PARP1 in HCMV infection may also result in a reduced antiviral response through a similar mechanism.

The HCMV genome is G-C rich and forms G4 structures within gene promoters, repeat-rich regions, and the oriLyt (114, 134, 135). G4 motifs are guanine rich sequences in DNA or RNA capable of forming highly stable and complex secondary structures called G4 tetrads, which can stack on top of each other, and are known to play roles in transcriptional regulation, obstruct progression of DNA replication and consequently stimulate recombination and genomic instability (136–138). These highly stable structures may require specialized host polymerases and helicases to resolve them and allow for replication. In KSHV, recruitment of the RecQ1 helicase to the oriLyt depends on the presence of a G4 motif; mutations within this motif reduce RecQ1 binding and impair vDNA replication (139, 140). These observations indicate that RecQ1-mediated unwinding of the G4 structure is critical for initiating KSHV DNA replication, likely by promoting further origin unwinding and enabling replication factor loading. Whether such a mechanism also contributes to HCMV replication remains to be investigated, as a G4 structure within essential region I (ER1) of the oriLyt was demonstrated to be necessary for the initiation of vDNA replication (114). The viral replication initiator proteins IE2 and UL84 directly interact with this structure, suggesting that it may function as a recognition site for specific viral and cellular replication factors (Fig. 4) (114). G4s have also been identified in other HCMV promoters and has both negative and positive regulatory roles. G4 formation within the UL35 and miR-US33 promoter regions has been shown to suppress promoter activity and thereby modulate transcription (135, 141). However, a G4 motif in the UL146 promoter region is necessary for transcription of the UL146–UL132 locus and IE2 mediates this transactivation upon binding to the G4 motif (142).

**Fig 5:**
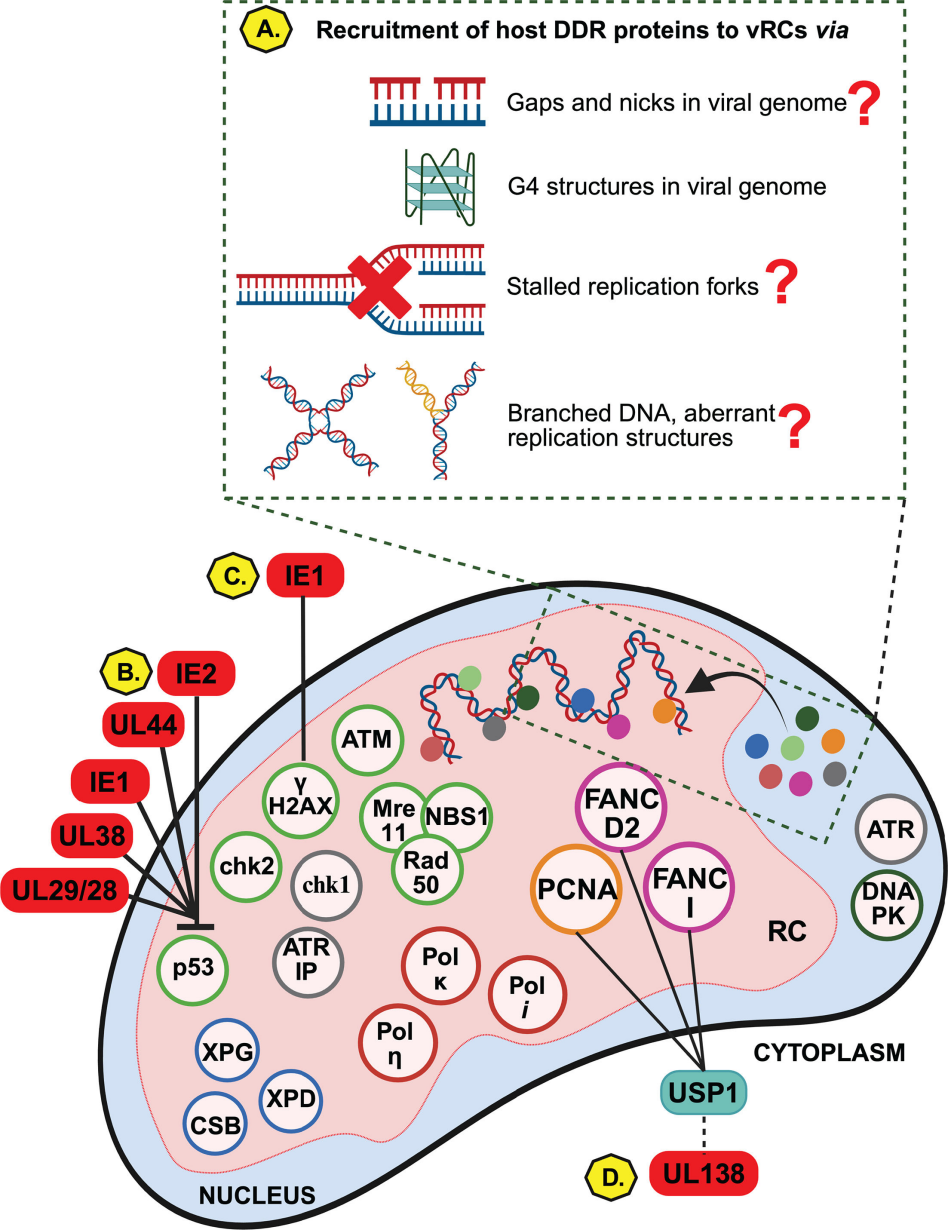
Recruitment of host DDR proteins to vRCs. Various host DDR factors are recruited to vRCs to regulate viral DNA synthesis, gene expression, and genome integrity. (**A**) Host factor recruitment to vRCs is partially due to the nature of the viral genome, which may contain nicks and gaps or complex DNA secondary structures like G4 structures. Additionally, genome synthesis can lead to stalled replication forks or aberrant replication structures containing branched DNA, which could also recruit host DDR proteins for resolution. (**B**) Many host proteins involved in ATM signaling localize to vRCs: ATM, Chk1, Chk2, H2AX, the MRN complex, and p53. Some DDR proteins, such as DNA-PK and ATR, are excluded from vRCs. IE2, UL44, IE1, UL38, and UL29/28 are implicated in the repression of p53 transcriptional activity. (**C**) IE1 expression is sufficient to induce serine 139 phosphorylation of H2AX (γH2AX), which is present within vRCs (red area inside nucleus). (**D**) A viral latency determinant, UL138, interacts with the deubiquitinating enzyme, USP1, which also localizes to vRCs. While the subcellular location of this UL138-USP1 interaction remains to be determined, UL138 influences ubiquitination of USP1 substrates that localize to vRCs: FANCD2, FANCI, and PCNA. These host proteins regulate TLS polymerases η, κ, and ι, all of which also localize to vRCs and promote viral genome integrity by canonical TLS function and also by non-canonical homology-directed mechanisms. UL138, through its host interactions, is important for directing HR mechanisms for high-fidelity synthesis of vDNA. Created in https://BioRender.com.

Localization of host proteins to RCs and activation of these proteins has been used as a potential indicator of their involvement in viral genome replication in several studies. The biological functions of these host factors on vDNA are poorly understood, and for many, their roles are solely inferred from their normal host functions. Isolation of protein on nascent DNA (iPOND) experiments in the laboratory-adapted strain, AD169, and low-passage strain, TB40/E, have identified host DDR factors associated with nascent vDNA, including DSB recognition and repair proteins, members of the cohesin complex, histones, and chromatin remodelers (143). Several components of the NER pathway, such as Cockaine’s syndrome B (CSB), xeroderma pigmentosum, complementation group D and G (XPD and XPG), are recruited to RCs (Fig. 5) (144). ATM signaling is activated upon HCMV infection (66) and its substrate, phosphorylated histone H2AX (γH2AX), localizes to RCs (67) (Fig. 5). A wide array of host DDR machinery, including the MRN complex, p53, chk1, chk2, and ATR interacting protein (ATRIP), are also localized to RCs (Fig. 5) (68). By contrast, DNA-PK and ATR, involved in NHEJ and replication stress response pathways, respectively, are excluded from RCs (68). These studies indicate that HCMV favors an ATM-mediated DDR and may be required for its replication as ATM inhibition results in decreased virus progeny (67, 68); however, how ATM contributes to virus replication remains elusive. Although DNA-PK is excluded from RCs at late time points during productive infection, it may have a role in circularizing viral genomes immediately upon entry into the nucleus, as seen in KSHV and HSV, for latency (55).

Despite encoding its own DNA polymerase, UL54, HCMV recruits specialized host translesion (TLS) polymerases to RCs. TLS polymerases play important roles in transversing DNA lesions and adducts that would otherwise result in replication fork stalling or restarting stalled forks. While TLS polymerases prevent fork stalling, allowing for the completion of DNA synthesis, and are critical to genome stability, they are error-prone and contribute to mutagenesis and cancer. In the context of HCMV infection, TLS polymerases contribute to viral genome integrity and diversity (145). Pol eta (η), iota (ι), and kappa (κ), which insert nucleotides across from a lesion, generate single nucleotide diversity on the viral genome. Their depletion decreases single nucleotide variants (SNVs) on the CMV genomes, indicative of their role in translesion repair. TLS polymerases are recruited to DNA by monoubiquitinated PCNA (mUb-PCNA), and during HCMV DNA synthesis, mUb-PCNA is required for generating SNVs (146). However, disruption of these TLS polymerases or pol zeta (ζ), a TLS pol important for extension past DNA lesions, also results in increased genomic rearrangements (inversions, deletions, and duplications) on the HCMV genome, which predominantly occur at regions of high G-C content and repetitive regions (145). Rearrangements are predominantly inversions between short (~20 bp) repeated sequences, which are indicative of HR and suggest a non-canonical (non-translesion) role for TLS polymerases in suppressing these types of HR events and/or DNA lesions that recruit HR factors. The approach of analyzing genomes reflects the importance of directly examining the effects of DDR pathway activation rather than simply cataloging activation or inactivation of DDR. TLS polymerases have known roles in a variety of non-translesion repair pathways; however, these activities are more poorly understood. While depletion of pol η, ι, and κ results in a modest increase in virus replication, depletion of the catalytic subunit of pol ζ (Rev 3L and Rev 7) or its scaffold (Rev1) decreases vDNA synthesis and production of progeny virus. Given the broad relocalization of host DDR factors, including p53 and specialized host repair polymerases, to viral RCs, it is likely that host mechanisms play a larger role in replication, recombination, or repair of viral genomes than previously appreciated (67, 144, 145).

It is also possible that recruitment or redirection of host DNA repair processes might come at the expense of host genome replication and fidelity (147), which could underlie oncomodulatory properties of HCMV. While cell division is arrested in productively infected fibroblasts, other cell types infected with HCMV, including CD34+ progenitors and monocytic cell lines, continue to divide, likely increasing their susceptibility to viral modulation of host DDR. HCMV IE1, as well as HSV1 ICP0, promote centromeric DNA amplification via TLS and thereby activate cGAS–STING signaling (148). HSV-1 ICP0 achieves these effects through direct degradation of centromeric proteins; however, the mechanism by which HCMV IE1 mediates this is not known. HCMV has also been shown to induce chromosomal breaks specifically on chromosome 1, which is linked to neuro-sensory hearing impairment (149, 150), a predominant clinical outcome of congenital CMV infection. A basement membrane protein, nidogen 1 (NID1), is encoded near one break site and is downregulated in HCMV infection. HCMV proteins, pp71 and UL35, target NID1 gene expression and protein turnover, respectively (151, 152). NID1 plays important roles in neuronal migration and excitability, and specifically regulates Schwann cell proliferation, migration, and myelin production, potentially underlying hearing deficiencies in congenital infection. Consistent with viral prioritization of genome maintenance, selective repair of UV-induced DNA damage in viral DNA over cellular DNA has also been reported in infected fibroblasts (144).

Components of the ubiquitin proteasome systems assemble into domains at the periphery of HCMV RCs, and ongoing proteasome activity is required for viral gene expression, particularly in early and late phases of infection (153). Further, UL35 associates with the ubiquitin-specific protease 7 (USP7) and components of the Cullin 4A (DCAF1, DDB1, and DDA1) E3 ubiquitin ligase complex, localizing these proteins to nuclear foci distinct from RCs (154). Ectopic expression of UL35 also activates a G2 cell cycle checkpoint in a DCAF1-dependent manner and induces γH2AX and p53 binding protein 1 (53BP1) foci, suggesting activation of a DNA damage response; however, the exact mechanism is unknown (154). Together, with tegument-derived pp71 (UL82), UL35 directs the proteasomal degradation of BCL-2-associated transcription factor, BCLAF1, a host restriction factor that represses transcription, promotes apoptosis, and limits HCMV gene expression and replication (155).

## VIRUS-HOST INTERACTIONS IN REGULATING HOST DDR FOR VIRUS REPLICATION

While the recruitment of numerous host DDR factors to vRCs underscores viral modulation of the DDR, the underlying mechanisms, consequences, and the strategies by which HCMV evades, co-opts, or redirects DDR activities remain incompletely understood. In the early points of infection, HCMV counters the antiviral activities of ND10s by targeting ND10 components for turnover. The viral tegument protein pp71 translocates to the nucleus ahead of the viral genome, where it binds hDaxx and induces its proteasomal degradation, thereby displacing ATRX and dismantling the hDaxx–ATRX complex to relieve repression at the MIEP (Fig. 3) (156). In addition, the viral immediate early one protein, IE1-72kDa, binds PML to prevent its small ubiquitin-like modifier conjugation (SUMOylation), disrupting the recruitment of SUMO-interacting proteins such as hDaxx and Sp100 (Fig. 3) (103, 157). Notably, ATRX remains nuclear and continues to moderate vDNA replication through its chromatin remodeling activity—a role that may ultimately benefit HCMV by preventing excessive genome amplification and ensuring efficient genome packaging and maturation (106). By contrast, in latency-permissive cells (e.g., CD34+ HPCs or monocytes), hDaxx is not eliminated to the same extent, and the ATRX–hDaxx complex remains intact to maintain repressive chromatin and silence IE transcription, promoting a latent state in these cells (158). Together, these distinct ND10-dependent and -independent roles of ATRX likely explain why HCMV does not directly target ATRX for degradation while selectively eliminating other ND10 constituents (106).

It is particularly noteworthy that this antagonism of ND10s by viral proteins may occur within a highly organized spatial context. For instance, HSV-1 genomes localize within distinct nuclear compartments known as vDNA-containing PML nuclear bodies (159) and are physically encapsulated within SUMOylated PML cages, which restrict viral gene expression and reactivation from latency (160). ND10s constitute pre-replication foci where viral transcription and genome synthesis are initiated to form RCs (112), and exogenous transgene arrays containing the MIEP have been shown to reside at the center of ND10s (161). In IE1-deficient HCMV infection, the host cell forms ring-like “PML cages” that can entrap vDNA and nuclear capsids (Fig. 3) (103, 162). The formation of these cages requires both IFN signaling and an active DNA damage response, the latter being orchestrated by the activation of ATM kinase, a key player in DNA repair (103, 162). These PML structures strongly associate with DNA damage markers; approximately 80% of PML cages colocalize with γH2AX, indicating that they arise at regions of persistent DNA damage (103). Notably, treatment of HCMVΔIE1-infected cells with an ATM inhibitor significantly reduces the number of giant PML-NBs or PML cages (103). This demonstrates that ATM-dependent DDR acts as an antiviral mechanism, which is then counteracted by viral proteins like IE1. Although IE1 dismantles ND10/PML cages, the virus may still exploit host DDR factors to facilitate viral genome maintenance and repair. For example, HCMV IE1 promotes phosphorylation of flap endonuclease 1 (FEN1), an HR-associated endonuclease that is involved in flap removal of Okazaki fragments during lagging strand synthesis and induction of DSBs to reinitiate stalled replication forks. FEN1 is required for optimal HCMV replication, suggesting involvement of HR-mediated repair of stalled replication forks during HCMV replication (163). It is not known whether IE1 targeting of FEN1 occurs in the context of early PML bodies, at vDNA structures engaged in HR-repair, or some other context.

UL138, a HCMV protein important for restricting replication for latency establishment, interacts with the deubiquitylation complex consisting of USP1 and the USP1-associated factor 1 (UAF1) complex to modulate interconnected host DNA repair pathways, which contribute to virus replication and genome integrity (164, 165). UAF1-USP1 regulates the monoubiquitination of proliferating cell nuclear antigen (PCNA), as well as FA complementation groups D2 and I (FANCD2 and FANCI), for recruitment of DDR factors, particularly TLS polymerases (Fig. 5). PCNA is restrictive to viral genome replication, and its modification by ubiquitination or SUMOylation at lysine 164 (K164) is important for this restriction (146). USP1, PCNA, and FANCD2 are recruited to viral RCs (146, 166). While USP1-PCNA/FANCD2/FANCI are important for viral genome integrity, our work defines roles for this complex beyond the regulation of canonical translesion synthesis (146). Interaction between USP1-PCNA/FANCD2/FANCI and UL138 protects viral genomes against large rearrangements, particularly inversions associated with G-C- and repeat-rich sequences (164). Interestingly, UL138 also protects the integrity of vDNA through additional pathways that appear to be independent of USP1-PCNA/FANCD2/FANCI and that may differentially impact UL versus US regions of the genome. Rearrangements associated with the loss of UL138 were predominantly inversions associated with repeat sequences, suggesting HR mechanisms. Beyond regulating the monoubiquitination of PCNA, FANCD2, and FANCI, USP1 is known to influence HR through interaction with RAD51AP1 (167). Additionally, UL138 interacts with epidermal growth factor receptor (EGFR) (168), which can translocate to the nucleus and phosphorylate DNA-PKcs, an important regulator of the NHEJ pathway (169). As UL138 sustains EGFR signaling in infection (168), UL138 may impact the activities of NHEJ and DSB repair pathways, but this awaits further investigation.

## VIRUS-HOST INTERACTIONS IN CONTROL OF THE CELL CYCLE THROUGH DDR PATHWAYS

DNA repair is inextricably linked to the cell cycle. Cell cycle checkpoints halt cell cycle progression to allow for DNA damage to be repaired. When damage is sensed by ATM or ATR, cells may arrest in G1, within S phase, or at G2. Extensive DNA damage that cannot be repaired will trigger apoptosis. HCMV induces a mitogenic response that stimulates a G1/S state primed for re-entry into the cell cycle but blocks progression through S phase, while vDNA replication can proceed with access to host replication/repair machinery and sufficient nucleotide pools (166, 170, 171). HCMV may co-opt components of the DDR pathways to partly achieve this G1/S phase arrest. Expression of IE1 induces activation of ATM, leading to downstream phosphorylation of p53 (66, 71), while IE2 stabilizes p53 by promoting degradation of MDM2, a ubiquitin ligase that targets p53 for proteasomal turnover (172). Presumably, phosphorylation and stabilization of p53 provide additional inhibition of cell cycle progression. Despite elevated phosphorylated p53 levels during infection, expression of p21—a cyclin-dependent kinase inhibitor and canonical readout of p53 transcriptional activity—is reduced, suggesting that p53-dependent transcription on the host genome is selectively inhibited in HCMV-infected cells (170, 173). p21 is additionally targeted by HCMV for proteasome- and calpain-dependent degradation early during infection (174). Consistent with this, IE2 partially represses p53-mediated transactivation by binding the histone acetyltransferase domains of the p53 coactivators p300 and CREB-binding protein (175, 176). Additional viral proteins, including IE1, UL44, UL29/28, and UL38, also interact with p53 and modulate its transcriptional activity on the host genome; however, the underlying mechanisms are unknown and appear to be distinct from IE2-mediated repression of p53 transcriptional activity (66, 177–179). Notably, viral genome replication is impaired in p53-deficient cells, and multiple viral genes are downregulated in the absence of p53, underscoring an essential role for p53 in productive infection (71, 180).

HCMV also drives the G1/S arrest by modulating E2F transcription factors and retinoblastoma (RB) family proteins. E2F (E2F 1–8) family of proteins are required for the expression of genes involved in G1/S transition and drive cell cycle progression, which are tightly controlled by the phosphorylation state of the RB family proteins (pRB, p107, p130). In early G1, hypophosphorylated RB family members bind E2F transcription factors and recruit chromatin-modifying enzymes to repress E2F-responsive promoters. pRB primarily associates with transcriptionally activating E2Fs (E2F1–3), whereas p107 and p130 preferentially bind the repressive E2Fs (E2F4 and E2F5). In G1/S transition, hyperphosphorylation of RB proteins by Cyclin D/CDK4/6 and Cyclin E/CDK2 results in dissociation of RB from DNA-bound RB-E2F complexes, leading to transcription of E2F-target genes involved in DNA replication, nucleotide biosynthesis, and cell cycle progression (181–183). Additionally, inactivation of RB proteins and deregulation of E2F1 is known to induce DSBs and contribute to genomic instability in normal diploid human cells (184). E2F1 is a direct target of ATM and ATR kinases and is stabilized through phosphorylation at ser31 by either kinase (185). E2F1 has been shown to have a nontranscriptional role in DNA repair—E2F1 accumulates at sites of DSBs and recruits repair factors such as NBS1, Rad51, and RPA (186). Several HCMV proteins are involved in the modulation of RB and E2F family of proteins. IE1 induces hyperphosphorylation of p107 and p130 while promoting activation of E2F1–3, thereby dismantling repressive E2F4–p107/p130 complexes and derepressing E2F-responsive promoters (187–192). Additionally, IE1 binding to p107 releases CyclinE/CDK2 from p107, allowing CyclinE/CDK2 kinase activity for G1 to early S phase cell cycle progression (193). IE2 reinforces this state by inducing phosphorylation of pRB and functioning as a transcriptional coactivator at E2F-responsive promoters (71, 194–196). Other HCMV proteins also contribute to this sustained inactivation of RB proteins; pp71 targets RB family of proteins for proteasomal degradation, and UL97 phosphorylates RB protein family members to relieve the repression on E2F family of transcription factors (197–199). Ectopic expression of IE proteins induces γH2AX foci on host genomes, and E2F1 was found to promote this accumulation both during infection and when IEs are ectopically expressed. E2F1 most likely increases the γH2AX foci via an ATM-mediated induction of DDR response, but the nature of DNA lesions that may be the source of the γH2AX foci is not clear. Based on these studies, it is evident that HCMV has co-opted several proteins to inactivate RB proteins to relieve the repression of E2F and to allow for transcription of genes involved in DNA synthesis and repair. Not surprisingly, E2F1 is required to efficiently replicate the HCMV genome (67).

Evidently, HCMV employs multiple viral proteins to manipulate components of the HR pathway to induce cell cycle arrest in G1 and early S phase. Additional regulators of the cell cycle, including cyclins and CDKs, are also extensively manipulated to promote a G1/S-like transition that sustains a prolonged S-phase after infection; however, discussion of these mechanisms is beyond the scope of this review.

## CONCLUSIONS AND FUTURE DIRECTIONS

DNA viruses (including phage) were the early workhorses of molecular biology. Now, it is widely accepted that DNA viruses have profound effects on cell cycle and DNA repair protein levels, localization, and function in both acute and latent infections (13, 55, 56, 200). However, much remains to be understood about how human DNA viruses modulate and co-opt DNA repair processes and the importance of this for controlling viral genome replication and, at least, in the case of herpesviruses, latency. Our work has also demonstrated roles for cellular DDR in maintaining genome fidelity, providing further support for the role of these host pathways in virus replication. Such work could reveal new therapeutic targets or strategies to combat HCMV persistence.

DNA damage and aberrant repair pose threats to human health from cancer to aging (201, 202). While the role of HCMV in cancer is unclear, HCMV is widely considered oncomodulatory (203–207). HCMV sequences have been detected in tumor tissues, and HCMV has been shown to alter metabolism and oncogenic properties of infected cells in ways that may impact the progression of oncogenesis. Viral manipulation of host DDR for replication or viral genome integrity may have important implications for the host cell and the integrity of its genome, particularly in cells that continue to divide after infection. It will be important going forward to understand if HCMV modulation of host DDR impacts host genome stability.

During DNA replication, numerous proteins act in a coordinated manner to ensure high-fidelity DNA synthesis, while also detecting and responding to damage. Repair of certain lesions, such as ICLs, requires the integrated activity of multiple repair pathways—including FA, TLS, HR, and NER (20)—in order to preserve genome integrity. Understanding how HCMV regulates these interconnected pathways and the consequences for the host and the host response is an important area for further work in understanding cellular DDR pathways where there are large gaps in our mechanistic understanding of these pathways. Analyzing the effects of host DDR manipulated by HCMV directly on vDNA (as well as on host DNA) is important to understanding the activity and functional consequences of the pathways targeted. As TLS polymerases were discovered a few decades ago, much remains to be discovered about their roles in human cells, particularly their roles beyond TLS. HCMV offers a strong tool for these investigations. The use of HCMV as a model for exploring how this nearly ubiquitous human virus co-opts host DDR has many advantages. HCMV primes and stimulates cells to promote HR pathways in G1 arrested cells, while preventing mitosis and cell division, thus eliminating confounding factors such as changes in mitotic chromatin compaction and chromosome segregation. Further, infection is efficient and provides a synchronized initiation of DNA synthesis in growth arrested cells that is amenable to and tolerant of knockdown of human DDR factors. Finally, the HCMV genome is easily modified, allowing for the roles of DNA *cis* elements and *trans* factors to be tested using recombinant viruses. Using HCMV as a model, we stand to learn how a common human pathogen co-opts, manipulates, and enhances host DNA repair processes.
